# Comparative Discriminative Performance of the HbA1c/HDL‐C Ratio, Stress Hyperglycemia Ratio, and ZJU Index for HOMA‐IR‐Defined Insulin Resistance in Patients With Type 2 Diabetes

**DOI:** 10.1155/ije/2925122

**Published:** 2026-10-21

**Authors:** Zhangna Zheng, Qianyi Li, Huijuan Zhang

**Affiliations:** ^1^ Department of Endocrinology The First Affiliated Hospital of Harbin Medical University Harbin China hrbmu.edu.cn

**Keywords:** HbA1c/HDL-C ratio, insulin resistance, stress hyperglycemia ratio, Type 2 diabetes, ZJU index

## Abstract

**Background:**

Routine assessment of insulin resistance (IR) remains challenging because fasting insulin measurements are not routinely available in many clinical settings. Several readily available metabolic indices, including the HbA1c/HDL‐C ratio, stress hyperglycemia ratio (SHR), and Zhejiang University (ZJU) index, have been associated with IR or related metabolic disturbances. However, their relative discriminative performance for HOMA‐IR‐defined IR in patients with established Type 2 diabetes has not been systematically compared. We aimed to compare their discriminative performance for HOMA‐IR‐defined IR and to assess whether ZJU provides incremental discrimination beyond conventional clinical and metabolic characteristics.

**Methods:**

This was a single‐center retrospective cross‐sectional study. We included 1000 hospitalized adults with established T2DM. HOMA‐IR was calculated from fasting plasma glucose and fasting insulin, and IR was operationally defined as HOMA‐IR ≥ 2.69. Multivariable logistic regression and restricted cubic spline analyses evaluated associations and nonlinear associations. Receiver operating characteristic analyses with paired DeLong tests compared discriminative performance. Additional analyses included TyG and TyG‐BMI, comparisons with individual ZJU components, nested‐model analysis, decision‐curve analysis, bootstrap internal validation, and medication‐adjusted sensitivity analyses.

**Results:**

All three indices were significantly associated with HOMA‐IR‐defined IR after multivariable adjustment. Compared with the lowest quartile, the adjusted odds ratios in the highest quartile were 3.16 (95% CI 1.95–5.18) for the HbA1c/HDL‐C ratio, 6.86 (4.11–11.84) for SHR, and 18.36 (10.21–34.79) for ZJU. Significant nonlinear associations were observed for all three indices. Among the evaluated composite indices, ZJU had the highest AUC (0.777, 95% CI 0.742–0.812), followed by TyG (0.749, 0.712–0.785), TyG‐BMI (0.733, 0.695–0.770), SHR (0.698, 0.659–0.738), and HbA1c/HDL‐C (0.615, 0.573–0.657). ZJU did not significantly outperform FPG alone (AUC 0.766; *p* = 0.509). Adding ZJU to the basic clinical model increased the AUC from 0.658 to 0.780 (ΔAUC = 0.123; *p* < 0.001), whereas adding ZJU to a more comprehensive model including its major metabolic components did not improve discrimination (0.806 vs. 0.807; *p* = 0.983).

**Conclusions:**

In hospitalized patients with established T2DM, ZJU demonstrated moderate discrimination for HOMA‐IR‐defined IR and provided incremental discrimination beyond basic clinical characteristics.

## 1. Introduction

Insulin resistance (IR) is a central pathophysiological feature of Type 2 diabetes mellitus (T2DM) and is closely linked to abnormalities in glucose, lipid, adipose, and hepatic metabolism [1, 2]. It contributes to progressive β‐cell dysfunction and impaired glucose homeostasis and is associated with cardiometabolic and metabolic liver disorders [1–3]. In patients with established T2DM, however, assessment of IR is primarily of research and metabolic characterization interest because direct measures are not routinely used as treatment targets. Accordingly, the present study focused on HOMA‐IR‐defined IR rather than implying direct measurement of physiological IR.

The hyperinsulinemic–euglycemic clamp is a reference method for assessing insulin sensitivity, but its complexity, cost, and intensive experimental requirements limit widespread clinical use [1, 4]. HOMA‐IR is widely used as a practical surrogate in epidemiological and clinical research, but it requires fasting insulin and is affected by insulin assay characteristics and population‐specific distributions [4, 5]. Because no universally accepted HOMA‐IR threshold exists, cut‐off values should be interpreted in the context of the population and assay methodology. These considerations have motivated investigation of simple indices based on routinely measured metabolic variables [4].

Several readily available metabolic indices have been investigated in relation to IR and related metabolic disturbances. The HbA1c/HDL‐C ratio combines chronic glycemic exposure with HDL‐C and has been associated with IR and metabolic abnormalities [6, 7]. The stress hyperglycemia ratio (SHR) relates glucose concentrations to HbA1c‐derived chronic glycemic status and has been studied extensively in acute cardiovascular and critical‐illness settings, with emerging evidence linking it to metabolic dysfunction [8–15]. The ZJU index combines BMI, FPG, TG, and the ALT/AST ratio and was originally developed for identifying fatty liver disease; subsequent work has reported associations with IR and diabetes risk [16–20]. The TyG index is another established surrogate based on fasting triglycerides (TG) and glucose, and TyG‐BMI extends this construct by incorporating adiposity [21, 22]. Because these indices summarize different metabolic domains, direct head‐to‐head comparison in patients with established T2DM may help clarify their relative discriminative performance.

Although previous studies have independently evaluated these indices, direct comparisons among HbA1c/HDL‐C, SHR, ZJU, TyG, and TyG‐BMI in patients with established T2DM remain limited. We therefore aimed to compare their discriminative performance for HOMA‐IR‐defined IR, evaluate nonlinear associations using restricted cubic splines (RCS), and determine whether ZJU provides incremental discrimination beyond conventional clinical and metabolic characteristics. We additionally compared ZJU with its individual components, explored potential net benefit using decision‐curve analysis, and performed bootstrap internal validation and medication‐adjusted sensitivity analyses. Because the study was cross‐sectional and involved hospitalized patients with established T2DM, we did not aim to establish a screening threshold or to demonstrate treatment utility.

## 2. Methods

### 2.1. Study Design and Study Population

This was a single‐center retrospective cross‐sectional study. A total of 1000 patients with T2DM who were hospitalized in the Department of Endocrinology of the First Affiliated Hospital of Harbin Medical University between January 2024 and December 2024 were consecutively enrolled (Figure 1). The study protocol was approved by the Institutional Ethics Committee of the hospital.

**FIGURE 1:**
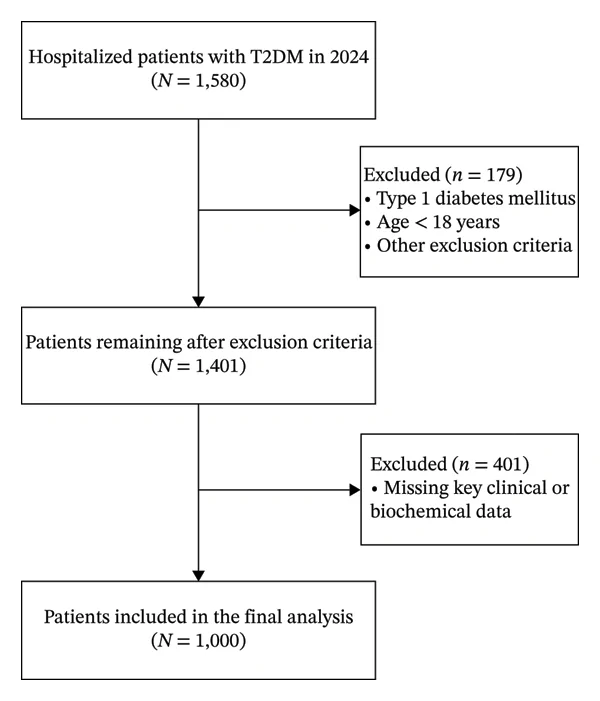
Study flow diagram.

T2DM was diagnosed according to the Chinese Guidelines for the Prevention and Treatment of Diabetes (2024 edition) [23]. Patients were diagnosed if they met any one of the following criteria: (1) typical symptoms of diabetes with a random plasma glucose ≥ 11.1 mmol/L; (2) fasting plasma glucose (FPG) ≥ 7.0 mmol/L; (3) 2‐h plasma glucose during an oral glucose tolerance test ≥ 11.1 mmol/L; or (4) HbA1c ≥ 6.5%. In the absence of typical symptoms, abnormal results were confirmed by repeat testing on a different day. Only patients with complete clinical and biochemical data were included. The cohort consisted of hospitalized patients with established T2DM and was not intended to represent community‐based screening populations or individuals with newly diagnosed diabetes.

#### 2.1.1. Exclusion Criteria

Patients were excluded if they met any of the following criteria:

1. Type 1 diabetes mellitus, gestational diabetes, or other specific types of diabetes;
2. Age < 18 years;
3. Incomplete baseline data;
4. Use of exogenous insulin or lipid‐lowering agents before admission;
5. History of pancreatitis or pancreatic surgery;
6. Acute stress conditions, such as diabetic ketoacidosis or hyperosmolar hyperglycemia state;
7. Malignancy;
8. Severe comorbid conditions affecting metabolic status, including primary glomerulonephritis, infectious diseases, drug‐induced kidney disease, pregnancy‐related conditions, vascular diseases, urinary system diseases, or hereditary diseases, or patients receiving renal replacement therapy (including dialysis or kidney transplantation).

### 2.2. Clinical and Laboratory Measurements

Demographic and clinical data were collected for all participants, including age, sex, duration of diabetes, history of hypertension, smoking status, and alcohol consumption. Smoking was defined as smoking at least one cigarette per day for more than one year and current smoking status. Alcohol consumption was defined as drinking alcohol (liquor, beer, or wine) at least two days per week for more than one year.

Physical examination included measurements of systolic blood pressure (SBP), diastolic blood pressure (DBP), height, and weight. Body mass index (BMI) was calculated as weight (kg) divided by height squared (m^2^).

Information on antidiabetic treatment at the time of hospitalization and during the hospital stay was recorded, including insulin, metformin, sulfonylureas, glinides, thiazolidinediones, α‐glucosidase inhibitors, DPP‐4 inhibitors, GLP‐1 receptor agonists, and SGLT2 inhibitors. Patients who had received exogenous insulin before admission were excluded because exogenous insulin could directly affect fasting insulin concentrations and consequently influence the calculation of HOMA‐IR. Therefore, the insulin treatment documented in the medication data refers to treatment at or after hospital admission and does not contradict the exclusion criterion for pre‐admission exogenous insulin use.

Laboratory measurements included glycated hemoglobin (HbA1c, %), fasting C‐peptide (ng/mL), fasting insulin (μU/mL), alanine aminotransferase (ALT, U/L), aspartate aminotransferase (AST, U/L), total cholesterol (TC, mmol/L), TG (mmol/L), low‐density lipoprotein cholesterol (LDL‐C, mmol/L), high‐density lipoprotein cholesterol (HDL‐C, mmol/L), serum creatinine (Cr, μmol/L), blood urea nitrogen (BUN, mmol/L), and uric acid (UA, μmol/L). After an overnight fast of at least 10 h, fasting blood samples were collected for the measurement of FPG and fasting insulin. Fasting insulin concentrations were measured in the Endocrinology Laboratory of the First Affiliated Hospital of Harbin Medical University using a chemiluminescence immunoassay on the MAGLUMI X3 analyzer (Snibe, China). HOMA1‐IR was calculated according to the original HOMA model proposed by Matthews et al. [5], using the following formula: HOMA1‐IR = FPG (mmol/L) × fasting insulin (μU/mL)/22.5. IR was defined as HOMA‐IR ≥ 2.69. This cut‐off was based on a large Chinese epidemiological study of 10,147 adults aged 25–74 years with normal glucose tolerance, in which 2.69 corresponded to the 75th percentile of the HOMA‐IR distribution [24]. This value was subsequently cited in the Chinese expert guidance on IR assessment as a reference cut‐off for defining IR in Chinese populations [25]. Given the lack of a universally accepted HOMA‐IR threshold and the influence of population characteristics and insulin assay methodology on HOMA‐IR values, the cut‐off of 2.69 was used as an operational study definition rather than a universal diagnostic threshold.

### 2.3. Definitions and Calculated Indices

The following indices were calculated using previously published definitions:

1. HbA1c/HDL‐C ratio = HbA1c (%)/HDL‐C (mmol/L) [6].
2. SHR = FPG (mmol/L)/[1.59 × HbA1c (%) − 2.59] [8].
3. ZJU index = FPG (mmol/L) + BMI (kg/m^2^) + TG (mmol/L) + 3 × ALT/AST ratio + 2 (if female) [16].
4. HOMA1‐IR = FPG (mmol/L) × fasting insulin (μU/mL)/22.5 [5].
5. TyG index = ln [TG (mg/dL) × FPG (mg/dL)/2] [22].
6. TyG‐BMI = TyG index × BMI [22].

### 2.4. Statistical Analysis

All statistical analyses were performed using R software (Version 4.4.3).

Continuous variables were tested for normality. Normally distributed variables were expressed as mean ± standard deviation (SD) and compared using independent‐samples *t*‐tests. Non‐normally distributed variables were presented as median (interquartile range, IQR) and compared using the Mann–Whitney *U* test. Categorical variables were expressed as numbers (percentages) and compared using the chi‐square test.

Multivariable logistic regression analyses were performed to evaluate the associations between the HbA1c/HDL‐C ratio, SHR, ZJU index, and HOMA‐IR‐defined IR. Three models were constructed. Model 1 was an unadjusted model. Model 2 was adjusted for age and sex, with BMI additionally included for the HbA1c/HDL‐C ratio and SHR. For the ZJU index, sex and BMI were not included in Model 2 because sex contributes directly to the ZJU formula (+2 for females) and BMI is an intrinsic component of the index. Model 3 was further adjusted for smoking status, alcohol consumption, diabetes duration, SBP, DBP, HDL‐C, LDL‐C, and serum creatinine (Cr), in addition to the covariates included in Model 2. For the ZJU index, sex and BMI remained excluded from Model 3 for the same component‐overlap rationale. Results were reported as odds ratios (ORs) with 95% confidence intervals (CIs). Because some covariates are directly embedded in the composite indices, the adjusted models were interpreted as association analyses and robustness analyses rather than as evidence of independent biological effects of the individual components.

RCS regression was applied based on fully adjusted models to explore potential nonlinear associations between each metabolic index and HOMA‐IR‐defined IR. Four knots were placed at the 5th, 35th, 65th, and 95th percentiles of each variable, with the median used as the reference value. Overall association and nonlinearity were assessed using the Wald test (*p* for overall and *p* for nonlinearity).

Receiver operating characteristic (ROC) curve analysis was used to evaluate the discriminative performance of HbA1c/HDL‐C, SHR, ZJU, TyG, TyG‐BMI, and the individual components of ZJU (BMI, FPG, TG, ALT, and AST) for HOMA‐IR‐defined IR. The area under the curve (AUC) and 95% CIs were calculated. Paired comparisons between correlated ROC curves were performed using the DeLong test, with false‐discovery‐rate adjustment for multiple pairwise comparisons where applicable.

Incremental Discrimination and Decision‐Curve Analysis: To assess whether ZJU provided information beyond conventional characteristics, nested logistic models were constructed. The basic clinical model included age, sex, diabetes duration, smoking, drinking, SBP, DBP, HbA1c, HDL‐C, LDL‐C, and creatinine; ZJU was then added. A more comprehensive clinical and metabolic model additionally included BMI, FPG, TG, ALT, and AST, followed by the addition of ZJU. Differences in AUC were assessed using paired DeLong tests. Decision‐curve analysis was performed to explore potential net benefit across threshold probabilities of 0.05–0.50. Because this was a cross‐sectional study without direct treatment decisions or clinical outcomes, DCA was considered exploratory and was not interpreted as evidence of clinical usefulness.

Internal Validation and Medication‐Adjusted Sensitivity Analysis: Internal validation was performed using 1000 bootstrap resamples to estimate optimism‐corrected AUCs. A medication‐adjusted sensitivity analysis additionally included major noninsulin glucose‐lowering therapies (metformin, α‐glucosidase inhibitors, DPP‐4 inhibitors, GLP‐1 receptor agonists, and SGLT2 inhibitors). Very sparse medication classes were not included in multivariable sensitivity models because of potential instability. Insulin use was not included because fasting insulin is a direct component of HOMA‐IR, and adjustment for insulin treatment could introduce circularity and overadjustment.

Subgroup analyses stratified by age, sex, and BMI were conducted, and interaction terms were introduced to assess potential effect modification. Given the number of subgroup comparisons and interaction tests, these analyses were considered exploratory and were interpreted cautiously, without treating isolated nominally significant interactions as definitive evidence of effect modification.

A two‐sided *p*‐value < 0.05 was considered statistically significant.

## 3. Results

### 3.1. Baseline Characteristics of the Study Population

A total of 1000 patients with T2DM were included. Among them, 239 patients (23.9%) were classified as non‐IR and 761 (76.1%) as HOMA‐IR‐defined IR. The baseline characteristics are presented in Table 1. Compared with the non‐IR group, patients with HOMA‐IR‐defined IR had higher DBP, FPG, HbA1c, TC, TG, LDL‐C, UA, fasting C‐peptide, fasting insulin, ALT, AST, γ‐GGT, BMI, HbA1c/HDL‐C, SHR, and ZJU (all *p* < 0.05). No significant differences were observed for age, height, duration of diabetes, SBP, Cr, BUN, sex, smoking, alcohol consumption, or hypertension prevalence (all *p* > 0.05).

**TABLE 1:** Clinical characteristics of the study participants.

	Total	Non‐IR	IR	*p*‐value
*N*	1000	239	761	
Age (years)	56.5 (48.0, 64.0)	57.0 (49.5, 64.0)	56.0 (48.0, 64.0)	0.354
Sex, *n* (%)	397 (39.7)	88 (36.8)	309 (40.6)	0.297
Smoke, *n* (%)	305 (30.5)	83 (34.7)	222 (29.1)	0.104
Drink, *n* (%)	308 (30.8)	79 (33.0)	229 (30.0)	0.387
Hypertension, *n* (%)	455 (45.5)	103 (43.1)	352 (46.2)	0.392
BMI (kg/m^2^)	25.4 (23.5, 27.7)	24.2 (22.1, 26.4)	25.9 (24.0, 28.0)	< 0.001
Diabetes duration (years)	10.0 (5.0, 16.0)	10.0 (6.7, 17.2)	10.0 (5.0, 16.0)	0.270
SBP (mmHg)	136 (123, 151)	135 (121, 148)	136.0 (124.0, 151.0)	0.145
DBP (mmHg)	85 (78, 93)	83 (76, 90)	86.0 (78.0, 95.0)	< 0.001
FPG (mg/dL)	151.9 (122.5196.0)	118.7 (100.5147.0)	163.2 (133.2207.2)	< 0.001
HbA1c (%)	8.4 (7.3, 9.7)	7.7 (6.7, 9.4)	8.5 (7.5, 9.9)	< 0.001
TC (mmol/L)	4.8 (4.0, 5.7)	4.6 (3.9, 5.5)	4.9 (4.1, 5.8)	0.007
TG (mmol/L)	1.7 (1.2, 2.7)	1.3 (0.9, 1.8)	1.9 (1.3, 3.1)	< 0.001
HDL‐C (mmol/L)	1.1 (0.9, 1.3)	1.1 (0.9, 1.3)	1.1 (0.9, 1.2)	< 0.001
LDL‐C (mmol/L)	2.9 (2.3, 3.6)	2.8 (2.2, 3.5)	3.0 (2.4, 3.7)	0.011
Cr (μmol/L)	65.0 (55.0, 76.5)	64.8 (54.5, 75.8)	65.0 (55.0, 77.0)	0.372
BUN (mmol/L)	5.7 (4.6, 6.8)	5.8 (4.7, 6.7)	5.6 (4.6, 6.8)	0.472
UA (μmol/L)	339.0 (281.8408.1)	313.9 (258.7385.2)	347.1 (289.0, 414.9)	< 0.001
Fasting C‐peptide (ng/mL)	2.4 (1.6, 3.3)	1.6 (1.1, 2.1)	2.7 (2.0, 3.6)	< 0.001
Fasting insulin (μU/mL)	11.3 (7.5, 16.5)	5.6 (2.9, 7.2)	13.4 (10.3, 18.7)	< 0.001
ALT (U/L)	20.6 (14.8, 31.8)	17.5 (13.2, 24.1)	22.1 (15.7, 34.6)	< 0.001
AST (U/L)	19.4 (15.6, 25.2)	18.0 (14.6, 22.9)	20.0 (15.9, 26.4)	< 0.001
γ‐GGT (U/L)	32.4 (21.8, 51.1)	24.9 (17.3, 41.2)	34.2 (23.6, 52.6)	< 0.001
HbA1c/HDL‐C	7.6 (6.0, 9.4)	6.7 (5.3, 8.7)	7.7 (6.2, 9.6)	< 0.001
SHR	0.8 (0.6, 0.9)	0.6 (0.5, 0.8)	0.8 (0.7, 0.9)	< 0.001
ZJU	41.1 (37.1, 45.4)	36.8 (33.8, 39.5)	42.5 (38.7, 46.3)	< 0.001

### 3.2. Multivariable Logistic Regression Analysis

Multivariable logistic regression analyses showed that higher levels of the HbA1c/HDL‐C ratio, SHR, and ZJU index were consistently associated with greater odds of HOMA‐IR‐defined IR across the sequentially adjusted models (Table 2). In Model 3, the odds of IR in the highest quartile (Q4) versus the lowest quartile (Q1) were 3.16 (95% CI: 1.95–5.18) for the HbA1c/HDL‐C ratio, 6.86 (95% CI: 4.11–11.84) for SHR, and 18.36 (95% CI: 10.21–34.79) for the ZJU index (all *p* for trend < 0.001). These associations remained evident after further adjustment for the prespecified covariates. For the ZJU index, sex and BMI were not included in the adjusted models because they are directly incorporated into the index. Given the cross‐sectional study design, these findings were interpreted as associations rather than causal or predictive effects.

**TABLE 2:** Associations of the HbA1c/HDL‐C ratio, SHR, and ZJU index with HOMA‐IR‐defined insulin resistance in multivariable logistic regression analyses.

Indices	Model					
	Model 1	*p*‐value	Model 2	*p*‐value	Model 3	*p*‐value
HbA1c/HDL						
Q1	1		1		1	
Q2	1.92 (1.30–2.87)		1.97 (1.33–2.94)		2.01 (1.30–3.13)	
Q3	2.09 (1.41–3.14)		2.20 (1.47–3.33)		2.06 (1.32–3.24)	
Q4	2.74 (1.81–4.20)	< 0.001^∗^	2.87 (1.87–4.45)	< 0.001^∗^	3.16 (1.95–5.18)	< 0.001^∗^
SHR						
Q1	1		1		1	
Q2	3.14 (2.13–4.70)		3.19 (2.16–4.78)		3.27 (2.11–5.12)	
Q3	3.31 (2.23–4.96)		3.39 (2.28–5.10)		3.40 (2.21–5.30)	
Q4	6.63 (4.19–10.78)	< 0.001^∗^	6.65 (4.20–10.82)	< 0.001^∗^	6.86 (4.11–11.84)	< 0.001^∗^
ZJU						
Q1	1		1		1	
Q2	3.26 (2.24–4.78)		3.37 (2.31–4.96)		3.53 (2.34–5.38)	
Q3	8.48 (5.40–13.71)		9.06 (5.73–14.75)		9.85 (6.00–16.68)	
Q4	13.06 (7.87–22.78)	< 0.001^∗^	15.46 (9.07–27.66)	< 0.001^∗^	18.36 (10.21–34.79)	< 0.001^∗^

*Note:* Model 1: Unadjusted model. Model 2: Adjusted for age and sex, with BMI additionally included for the HbA1c/HDL‐C ratio and SHR. For the ZJU index, sex and BMI were not included because sex contributes directly to the ZJU formula (+2 for females), and BMI is an intrinsic component of the ZJU index. Model 3: Further adjusted for smoking status, alcohol consumption, diabetes duration, systolic blood pressure (SBP), diastolic blood pressure (DBP), high‐density lipoprotein cholesterol (HDL‐C), low‐density lipoprotein cholesterol (LDL‐C), and serum creatinine (Cr), in addition to the covariates included in Model 2. For the ZJU index, sex and BMI were not included for the same component‐overlap rationale described above.

^∗^ *p* for trend.

### 3.3. RCS Analysis

RCS analyses revealed significant nonlinear associations between all three metabolic indices and HOMA‐IR‐defined IR (all *p* for overall < 0.001; all *p* for nonlinearity < 0.05) (Figures 2, 3, and 4). The HbA1c/HDL‐C ratio demonstrated a threshold‐like nonlinear pattern, SHR showed a continuously increasing nonlinear pattern, and ZJU showed an S‐shaped nonlinear association with increasing odds of HOMA‐IR‐defined IR at higher values. These patterns were interpreted as nonlinear associations.

**FIGURE 2:**
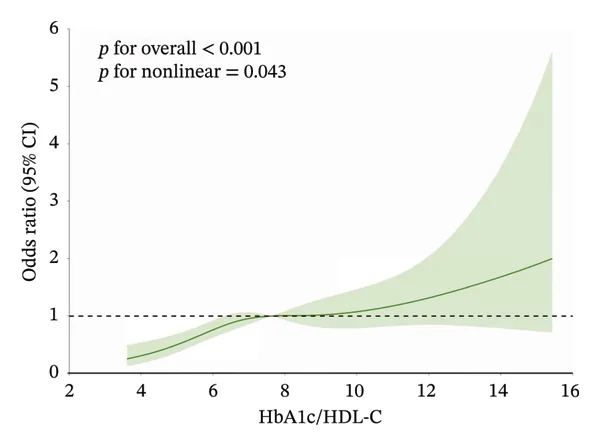
HbA1c/HDL‐C.

**FIGURE 3:**
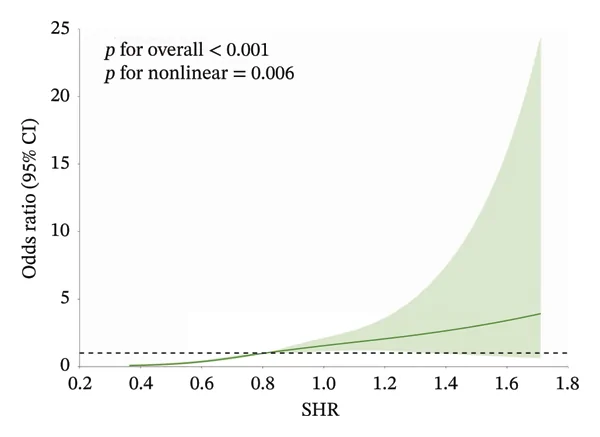
SHR.

**FIGURE 4:**
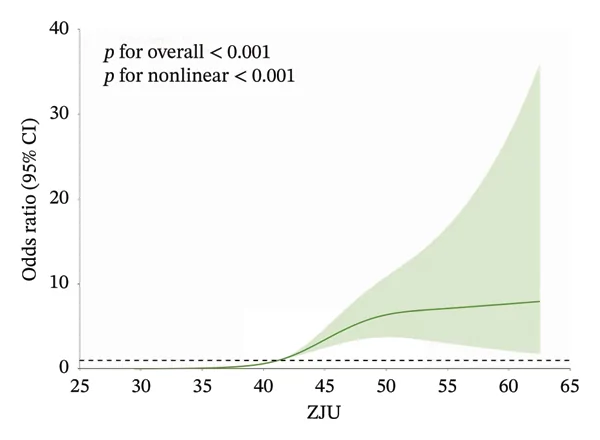
ZJU.

### 3.4. ROC Curve Analysis

ROC curve analysis (Figure 5) showed that ZJU had the highest AUC among the evaluated composite indices (0.777, 95% CI 0.742–0.812), followed by TyG (0.749, 0.712–0.785), TyG‐BMI (0.733, 0.695–0.770), SHR (0.698, 0.659–0.738), and HbA1c/HDL‐C (0.615, 0.573–0.657). ZJU did not significantly outperform FPG alone (AUC 0.766; *p* = 0.509). Sex‐stratified AUCs are shown in Table 3; these analyses were considered exploratory.

**FIGURE 5:**
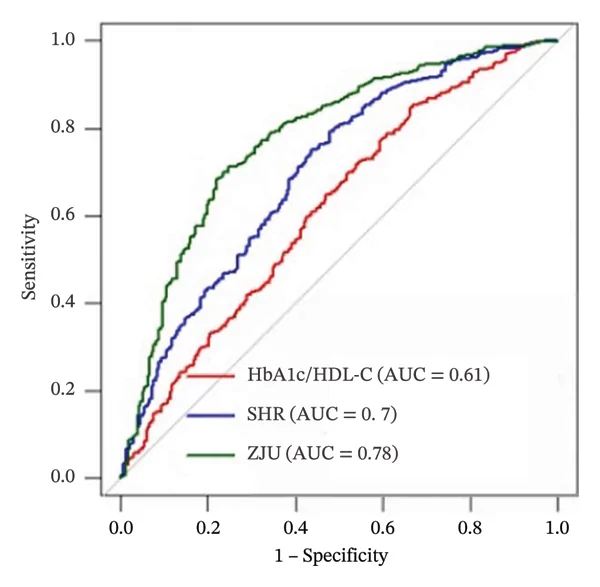
ROC.

**TABLE 3:** Receiver operating characteristic (ROC) curve analysis by sex.

	Sex	AUC
HbA1c/HDL‐C	Male	0.588
SHR	Male	0.697
ZJU	Male	0.770
HbA1c/HDL‐C	Female	0.665
SHR	Female	0.698
ZJU	Female	0.779

### 3.5. DeLong’s Test

Paired DeLong analyses showed that ZJU had significantly higher AUCs than TyG‐BMI, SHR, HbA1c/HDL‐C, BMI, TG, ALT, and AST. The difference between ZJU and TyG was modest and borderline after multiple‐comparison adjustment (*p* = 0.0483). ZJU did not significantly outperform FPG (*p* = 0.509). Detailed pairwise comparisons are provided directly in Table 4.

**TABLE 4:** Receiver operating characteristic (ROC) analysis and pairwise DeLong comparisons of metabolic indices for HOMA‐IR‐defined insulin resistance.

Indicator	AUC	95% CI	Cut‐off	Sensitivity	Specificity	Youden	FDR‐adjusted DeLong *p*‐value vs. ZJU
HbA1c/HDL‐C	0.615	0.573–0.657	5.615	0.852	0.332	0.184	< 0.001
SHR	0.698	0.659–0.738	0.704	0.792	0.521	0.313	< 0.001
ZJU	0.777	0.742–0.812	39.957	0.685	0.782	0.466	—
TyG	0.749	0.712–0.785	9.074	0.760	0.664	0.423	0.048
TyG‐BMI	0.733	0.695–0.770	227.089	0.727	0.655	0.382	< 0.001
BMI	0.648	0.607–0.688	24.250	0.717	0.513	0.230	< 0.001
FPG	0.766	0.730–0.801	6.975	0.821	0.601	0.422	0.509
TG	0.674	0.636–0.713	1.885	0.509	0.773	0.282	< 0.001
ALT	0.631	0.591–0.671	25.050	0.430	0.782	0.211	< 0.001
AST	0.580	0.539–0.621	20.250	0.490	0.639	0.129	< 0.001

### 3.6. Incremental Discrimination, Decision‐Curve Analysis, and Internal Validation

In the basic clinical model (Table 5), the AUC was 0.658 (95% CI 0.618–0.697) and increased to 0.780 (0.746–0.814) after adding ZJU (ΔAUC = 0.123; paired DeLong *p* < 0.001). In the more comprehensive clinical and metabolic model, the AUC was 0.807 (0.774–0.839), and adding ZJU did not improve discrimination (AUC 0.806; paired DeLong *p* = 0.983). Decision‐curve analysis suggested a higher net benefit after adding ZJU to the basic clinical model across threshold probabilities of approximately 0.22–0.50; this was considered exploratory and was not interpreted as evidence of clinical usefulness.

**TABLE 5:** Incremental discrimination of ZJU in nested models.

Model	*N*	AUC	95% CI	ΔAUC	DeLong *p*
Basic clinical model	1000	0.658	0.618–0.697	—	—
Basic clinical model + ZJU	1000	0.780	0.746–0.814	+0.123	< 0.001
Comprehensive clinical model	1000	0.807	0.774–0.839	—	—
Comprehensive model + ZJU	1000	0.806	0.774–0.839	−0.000	0.983

Bootstrap internal validation (Supporting Table S1) with 1000 resamples yielded optimism‐corrected AUCs of 0.631 for the basic clinical model and 0.766 for the model including ZJU. For the more comprehensive model, the corresponding optimism‐corrected AUCs were 0.789 without ZJU and 0.787 with ZJU, supporting the overall pattern observed in the primary analysis.

### 3.7. Medication‐Adjusted Sensitivity Analysis

After adjustment for major noninsulin glucose‐lowering therapies, the AUC increased from 0.730 to 0.795 after adding ZJU to the basic clinical model (paired DeLong *p* ≤ 0.001) (Supporting Table S2). In contrast, adding ZJU to the comprehensive model did not improve discrimination (AUC 0.824 vs. 0.824; *p* = 0.917). Bootstrap optimism‐corrected AUCs were 0.702 and 0.775 for the medication‐adjusted basic models without and with ZJU, respectively, and 0.801 and 0.800 for the corresponding comprehensive models.

### 3.8. Subgroup Analysis

Subgroup analyses stratified by sex, age, and BMI are shown in Figures 6, 7, and 8. A nominally significant interaction was observed between sex and the HbA1c/HDL‐C ratio (*p* for interaction = 0.044) and between BMI and ZJU (*p* for interaction = 0.028). These subgroup findings should be interpreted cautiously because multiple interaction tests were performed and no adjustment for multiplicity was prespecified. The analyses are therefore considered hypothesis‐generating rather than definitive evidence of effect modification.

**FIGURE 6:**
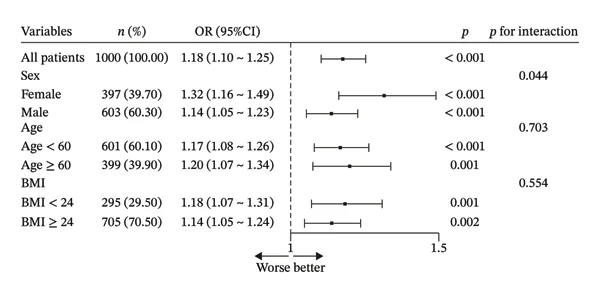
HbA1c/HDL‐C subgroup analyses.

**FIGURE 7:**
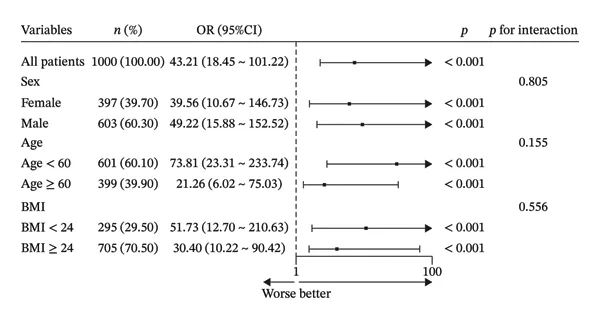
SHR subgroup analyses.

**FIGURE 8:**
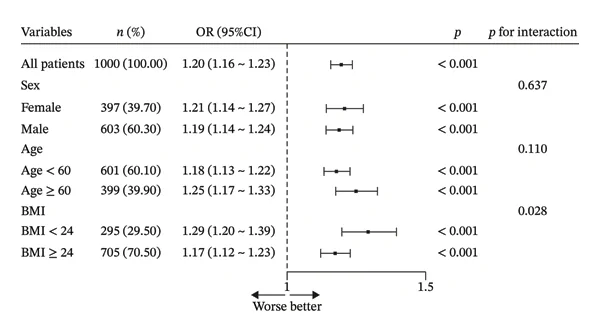
ZJU subgroup analyses.

For the ZJU index, a significant interaction was observed with BMI (*p* for interaction = 0.028), indicating a stronger association in patients with BMI < 24 kg/m^2^ (OR = 1.29, 95% CI: 1.20–1.39) compared with those with BMI ≥ 24 kg/m^2^ (OR = 1.17, 95% CI: 1.12–1.23). Overall, the ZJU index showed consistent associations with IR across most subgroups.

## 4. Discussion

### 4.1. Principal Findings

In this single‐center study of hospitalized patients with established T2DM, higher HbA1c/HDL‐C ratio, SHR, and Zhejiang University (ZJU) index were consistently associated with HOMA‐IR‐defined IR. Among the evaluated composite indices, ZJU showed the highest discrimination, although its AUC was not significantly higher than that of FPG alone. Importantly, the incremental analyses provided additional insight into the nature of this apparent advantage. Adding ZJU to the basic clinical model substantially improved discrimination, whereas incorporation of ZJU into a more comprehensive model containing its major metabolic components did not provide additional discriminatory information. These findings suggest that the relatively strong performance of ZJU primarily reflects the integration of several interrelated metabolic characteristics rather than a substantial amount of information independent of its individual components.

This interpretation is consistent with the current understanding of IR as a systemic metabolic disorder involving coordinated abnormalities in skeletal muscle, liver, and adipose tissue rather than a defect confined to a single metabolic pathway [1, 2]. Impaired insulin‐mediated glucose disposal in skeletal muscle, inadequate suppression of hepatic glucose production, and increased adipose tissue lipolysis can occur simultaneously and interact through circulating substrates and tissue‐to‐tissue metabolic communication [1, 2]. Therefore, a composite index that incorporates several metabolic domains may capture a broader insulin‐resistant phenotype than an index reflecting only one aspect of glucose or lipid metabolism.

The present findings also highlight an important distinction between discrimination and biological specificity. Although ZJU showed the highest AUC among the evaluated composite indices, the absence of additional discrimination after inclusion of its major components indicates that its performance should not be interpreted as evidence of a unique biomarker of physiological IR. Rather, ZJU appears to provide a concise summary of information already distributed across routinely measured anthropometric and biochemical variables. This distinction is particularly relevant when interpreting composite surrogate indices, because a higher discriminative performance does not necessarily indicate that the composite index contains independent biological information beyond its components [4].

### 4.2. HbA1c/HDL‐C Ratio and IR

The HbA1c/HDL‐C ratio combines chronic glycemic exposure with HDL‐C and therefore represents two interrelated metabolic domains. In the present study, a higher HbA1c/HDL‐C ratio was consistently associated with HOMA‐IR‐defined IR, supporting previous observations linking this composite measure with metabolic abnormalities [6, 7].

From a biological perspective, chronic IR impairs insulin‐mediated glucose disposal and reduces the ability of insulin to suppress hepatic glucose production, contributing to persistent hyperglycemia and increased HbA1c levels [1, 2]. At the same time, IR is accompanied by increased adipose tissue lipolysis and altered lipid flux, which can promote hepatic TG‐rich lipoprotein production and changes in HDL metabolism [1, 2, 7]. Thus, the HbA1c/HDL‐C ratio may reflect the coexistence of chronic glycemic burden and an adverse lipid phenotype associated with IR.

However, the discriminatory performance of the HbA1c/HDL‐C ratio in our cohort was lower than that of ZJU, TyG, and TyG‐BMI. One possible explanation is that HbA1c/HDL‐C captures a narrower metabolic spectrum, primarily representing chronic glycemic exposure and HDL‐related lipid metabolism, whereas ZJU additionally incorporates adiposity, fasting glucose, TG metabolism, and hepatic biochemical characteristics. The present results therefore suggest that HbA1c/HDL‐C may be useful as a simple marker of an insulin‐resistant metabolic phenotype but may provide less comprehensive metabolic information than indices incorporating multiple domains.

An additional consideration is mathematical coupling. Because HDL‐C is directly embedded in the HbA1c/HDL‐C ratio, adjustment for HDL‐C in multivariable analyses may introduce overlap between the exposure and covariate. Therefore, the adjusted association should be interpreted as an association of the composite phenotype rather than evidence that the ratio exerts an independent biological effect.

### 4.3. SHR and IR

SHR provides a different metabolic perspective from conventional glucose measures by expressing the current glucose concentration relative to the background glycemic state estimated from HbA1c. The original description of SHR emphasized its ability to quantify relative rather than absolute hyperglycemia by accounting for chronic glycemic exposure [8]. Subsequent studies have primarily investigated SHR in acute cardiovascular and critical‐illness settings, where relative hyperglycemia has been associated with adverse clinical outcomes [8–15].

In the present study of hospitalized patients with established T2DM, SHR was positively associated with HOMA‐IR‐defined IR and showed moderate discrimination. From an endocrine perspective, IR reduces insulin‐mediated glucose disposal and impairs the suppression of hepatic glucose production, thereby increasing the potential discrepancy between contemporaneous glucose concentrations and an individual’s chronic glycemic background [1, 2]. In addition, acute physiological stress may activate counter‐regulatory pathways involving catecholamines, cortisol, glucagon, and growth hormone, which can further increase hepatic glucose production and impair glucose utilization [8]. Thus, a higher SHR may capture both the underlying metabolic susceptibility to relative hyperglycemia and the acute metabolic perturbations associated with hospitalization.

The lower AUC of SHR compared with ZJU may reflect the different biological constructs represented by these indices. SHR primarily characterizes the relationship between current and chronic glycemia, whereas ZJU incorporates several additional metabolic domains, including adiposity, TG metabolism, and hepatic biochemical characteristics. Importantly, because the present cohort consisted of hospitalized patients, SHR should not be interpreted as a specific marker of chronic IR. Hospitalization itself may introduce acute metabolic stress that influences the current glucose concentration. Therefore, the observed association should be regarded as an association between relative hyperglycemia and HOMA‐IR‐defined IR in this specific clinical setting rather than evidence that SHR directly measures chronic insulin sensitivity.

### 4.4. Biological Basis for the Performance of the ZJU Index

The relatively strong discrimination of the ZJU index is biologically plausible because its components represent several interconnected domains of insulin‐resistant metabolism. The ZJU index was originally developed from BMI, FPG, TG, and the ALT/AST ratio as a model for identifying nonalcoholic fatty liver disease (NAFLD) in a Chinese population [23]. Subsequent evidence demonstrated that higher ZJU values were associated with higher fasting insulin concentrations and HOMA‐IR and that the index could help identify IR in the Chinese general population [26]. More recent population‐based studies have further linked ZJU with diabetes incidence and metabolic liver disease [27–29]. Together, these findings support the interpretation of ZJU as an integrative marker of metabolic dysfunction rather than a marker of a single physiological pathway.

BMI provides information about adiposity. Excess adiposity, particularly when accompanied by adipose tissue dysfunction, can increase free fatty acid release and alter adipokine and inflammatory signaling. Increased fatty acid flux to the liver and skeletal muscle may promote ectopic lipid accumulation and interfere with insulin signaling, thereby contributing to both hepatic and peripheral IR [1, 2]. Importantly, the reported association between ZJU and metabolic liver disease in nonobese populations suggests that the information captured by ZJU is not simply equivalent to obesity status and may reflect additional metabolic abnormalities beyond adiposity alone [29].

FPG captures an important consequence of impaired insulin‐mediated glucose regulation. In hepatic IR, insulin becomes less effective at suppressing gluconeogenesis and glycogenolysis, resulting in increased endogenous glucose production and higher fasting glucose concentrations [1, 2]. Thus, the glucose component of ZJU provides information from the glycemic domain that complements the adiposity component.

TG represents another closely related component of insulin‐resistant metabolism. In the insulin‐resistant state, impaired suppression of adipose tissue lipolysis increases free fatty acid delivery to the liver, which can promote hepatic TG synthesis and secretion of TG‐rich lipoproteins [1, 2]. Lipid accumulation within skeletal muscle and liver may further interfere with insulin signaling through lipid‐derived intermediates [2]. Consequently, elevated TG may provide a readily available biochemical signal of altered lipid flux and insulin‐resistant metabolism.

The ALT/AST ratio may provide an additional indirect signal of hepatic metabolic disturbance. Hepatic IR is closely linked to abnormal hepatic lipid accumulation and altered glucose and lipid metabolism [3]. Previous studies have demonstrated associations between ZJU and NAFLD in the general Chinese population [23, 28], and longitudinal evidence has suggested an association between ZJU and subsequent development of NAFLD even among nonobese individuals [29]. However, liver enzymes are not specific measures of hepatic insulin sensitivity or hepatic steatosis. Therefore, the hepatic component of ZJU should be interpreted as an indirect representation of hepatic metabolic characteristics rather than a direct measurement of hepatic insulin action.

Taken together, BMI, FPG, TG, and the ALT/AST ratio represent partially overlapping but biologically complementary aspects of metabolic dysfunction. Their integration into ZJU may therefore explain its relatively strong discrimination in our cohort. The association of ZJU with IR [26], diabetes incidence [27], and metabolic liver disease [23, 28, 29] across different populations further supports its role as an integrative metabolic marker. Importantly, however, these associations do not establish that ZJU measures insulin action directly or that it provides biological information independent of its individual components.

### 4.5. Composite Indices Versus Their Individual Components

An important finding of the present study is that the apparent advantage of a composite index should be distinguished from information already available from its individual components. Adding ZJU to the basic clinical model increased the AUC from 0.658 to 0.780, indicating that ZJU summarized metabolic information that was not fully represented by the basic clinical characteristics. However, when BMI, FPG, TG, ALT, and AST were incorporated into the more comprehensive clinical/metabolic model, adding ZJU did not further improve discrimination (AUC 0.807 versus 0.806, *p* = 0.983). This pattern indicates that the information captured by ZJU is largely represented by its constituent metabolic variables.

This distinction is important when interpreting surrogate indices of IR. Composite measures can improve convenience by reducing several correlated metabolic characteristics to a single numerical value, but combining variables does not necessarily create new biological information. The relatively high AUC of a composite index may therefore reflect its ability to integrate several related manifestations of metabolic dysfunction rather than superiority as an independent marker of insulin action [4].

The same principle helps explain the differences observed among the indices evaluated in this study. HbA1c/HDL‐C emphasizes chronic glycemic exposure and HDL‐C, whereas SHR characterizes relative hyperglycemia. TyG incorporates fasting TG and glucose, while TyG‐BMI additionally incorporates adiposity [21, 22]. ZJU extends this multidimensional representation by incorporating adiposity, glycemia, TG metabolism, and hepatic biochemical characteristics [16, 17]. Consequently, differences in discrimination among these indices may partly reflect the breadth of metabolic domains represented by each formula and the degree of overlap among their components.

The findings therefore support a conceptual distinction between an integrative surrogate and an independent biomarker. ZJU may be useful because it summarizes several metabolic characteristics into a single score, but the present data do not support the interpretation that ZJU contains substantial information beyond those characteristics. This interpretation is also consistent with the absence of incremental discrimination after incorporation of the major ZJU components.

### 4.6. Clinical and Biological Relevance

The clinical relevance of these findings should be considered in the context of established T2DM. HOMA‐IR‐defined IR is primarily a research phenotype in this population rather than a routinely used treatment target, and surrogate indices should therefore not automatically be interpreted as tools for treatment selection or clinical decision‐making [4]. The present study was designed to characterize associations with HOMA‐IR‐defined IR rather than to establish a diagnostic threshold or demonstrate that index‐guided management improves clinical outcomes.

Nevertheless, the findings may have practical relevance for metabolic phenotyping. ZJU can be calculated from routinely available anthropometric and biochemical variables and does not require fasting insulin measurement. In settings where direct assessment of insulin sensitivity is impractical, such an index may provide a convenient summary of the overall metabolic characteristics associated with IR. Its practical value therefore lies primarily in integration and accessibility rather than in providing information that is biologically independent of its components.

This distinction is particularly relevant in established T2DM, where many metabolic abnormalities coexist and are biologically interconnected. A composite index may facilitate rapid characterization of this metabolic phenotype, but its interpretation should remain complementary to conventional clinical and biochemical assessment. The present findings do not demonstrate that ZJU should replace FPG, TG, BMI, liver enzymes, or established measures of IR.

Furthermore, although ZJU showed relatively favorable discrimination and exploratory decision‐curve characteristics in this cohort, these findings do not establish clinical utility in the sense of improving patient outcomes. The exploratory threshold identified in the present dataset should therefore not be regarded as a validated diagnostic cut‐off. External validation in independent populations, preferably including nonhospitalized patients, newly diagnosed T2DM, and different ethnic or clinical settings, is necessary before any threshold‐based application can be considered.

### 4.7. Strengths and Limitations

The present study has several strengths. First, multiple readily available metabolic indices were directly compared within the same cohort of patients with established T2DM. Second, paired DeLong comparisons, nested‐model analyses, decision‐curve analysis, bootstrap internal validation, and medication‐adjusted sensitivity analyses were used to assess discrimination and robustness from complementary perspectives. Third, the study explicitly examined whether the apparent performance of ZJU persisted beyond its individual metabolic components, allowing the distinction between integrated information and incremental information.

Several limitations should also be acknowledged. First, the cross‐sectional design prevents causal inference and does not establish that any index predicts future IR or clinical outcomes. Second, the study was conducted at a single center among hospitalized patients with established T2DM, which limits generalizability to community‐based populations, screening settings, newly diagnosed diabetes, or earlier stages of T2DM. Third, HOMA‐IR is a surrogate measure rather than a direct measure of physiological IR, and the cut‐off of 2.69 was used as a study‐specific operational definition rather than a universally accepted diagnostic threshold. Fourth, the ZJU threshold and apparent discrimination were derived and evaluated within the same cohort; although bootstrap internal validation was performed, optimism cannot be completely excluded, and external validation remains necessary. Fifth, some covariates overlap with components of the investigated indices, creating potential mathematical coupling or overadjustment and limiting the interpretation of adjusted coefficients. Finally, although medication‐adjusted sensitivity analyses were performed, residual confounding and confounding by indication cannot be completely excluded.

## 5. Conclusion

In hospitalized patients with established T2DM, the HbA1c/HDL‐C ratio, SHR, and ZJU index were associated with HOMA‐IR‐defined IR, and ZJU demonstrated the highest discrimination among the evaluated composite indices. ZJU improved discrimination beyond basic clinical characteristics, but not beyond its major metabolic components. These findings suggest that ZJU may serve as an integrative metabolic index for research‐oriented assessment of HOMA‐IR‐defined IR in this specific population, rather than as a validated screening or treatment‐guidance tool. The exploratory threshold of approximately 40 requires external validation before any clinical application can be considered.

## Author Contributions

Zhangna Zheng: collecting information and data and writing–original draft. Qianyi Li: review and editing. Huijuan Zhang: supervision and review and editing.

## Funding

The authors did not receive support from any organization for the submitted work.

## Disclosure

All authors read and approved the final manuscript.

## Ethics Statement

This study was approved by the Ethics Committee of the First Affiliated Hospital of Harbin Medical University (Approval No. 2026202) and was conducted in accordance with the Declaration of Helsinki and its subsequent amendments. The requirement for written informed consent was waived by the Ethics Committee because of the retrospective nature of the study.

## Conflicts of Interest

The authors declare no conflicts of interest.

## Supporting Information

Additional supporting information can be found online in the Supporting Information section.

## Supporting information


**Supporting Information** Bootstrap internal validation of model discrimination and Medication‐adjusted sensitivity analysis in Supporting Table S1‐S2.

## Data Availability

The data that support the findings of this study are available from the corresponding author upon reasonable request.
