# Supplementary material for: Comparative Discriminative Performance of the HbA1c/HDL‐C Ratio, Stress Hyperglycemia Ratio, and ZJU Index for HOMA‐IR‐Defined Insulin Resistance in Patients With Type 2 Diabetes
Source: Int J Endocrinol. 2026 Oct 21;2026:2925122. doi: 10.1155/ije/2925122 (PMC13686360; doi:10.1155/ije/2925122)
Supplement: Supplementary file 1 [file IJE-2026-2925122-s001.docx]

**Supplementary Table S1. Bootstrap internal validation of model discrimination.**

| Model | Apparent AUC | Mean optimism | Optimism-corrected AUC |
| --- | --- | --- | --- |
| Basic clinical model | 0.658 | 0.027 | 0.631 |
| Basic clinical model + ZJU | 0.780 | 0.015 | 0.766 |
| Comprehensive clinical model | 0.807 | 0.018 | 0.789 |
| Comprehensive model + ZJU | 0.806 | 0.019 | 0.787 |

**Supplementary Table S2. Medication-adjusted sensitivity analysis.**

| Model | AUC | 95% CI | ΔAUC vs corresponding base model | DeLong P | Optimism-corrected AUC |
| --- | --- | --- | --- | --- | --- |
| Medication-adjusted basic model | 0.730 | 0.695–0.765 | — | — | 0.702 |
| Medication-adjusted basic model + ZJU | 0.795 | 0.763–0.827 | +0.065 | <0.001 | 0.775 |
| Medication-adjusted comprehensive model | 0.824 | 0.794–0.855 | — | — | 0.801 |
| Medication-adjusted comprehensive model + ZJU | 0.824 | 0.793–0.854 | −0.0004 | 0.917 | 0.800 |
